# Poikiloderma With Neutropenia and Mastocytosis: A Case Report and a Review of Dermatological Signs

**DOI:** 10.3389/fmed.2021.680363

**Published:** 2021-06-10

**Authors:** Vincenzo Piccolo, Teresa Russo, Daniela Di Pinto, Elvira Pota, Martina Di Martino, Giulio Piluso, Andrea Ronchi, Giuseppe Argenziano, Eugenia Veronica Di Brizzi, Claudia Santoro

**Affiliations:** ^1^Dermatology Unit, University of Campania “Luigi Vanvitelli”, Naples, Italy; ^2^Department of Women and Child Health and General and Specialized Surgery, University of Campania “Luigi Vanvitelli”, Naples, Italy; ^3^Department of Precision Medicine, University of Campania “Luigi Vanvitelli”, Naples, Italy; ^4^Anatomic Pathology Unit, University of Campania “Luigi Vanvitelli”, Naples, Italy; ^5^Department of Physical and Mental Health, and Preventive Medicine, University of Campania “Luigi Vanvitelli”, Naples, Italy

**Keywords:** mastocytosis, skin cancer, USB1, COVID-19, poikiloderma with neutropenia

## Abstract

Poikiloderma with neutropenia (PN) is a very rare genetic disorder mainly characterized by poikiloderma and congenital neutropenia, which explains the recurrence of respiratory infections and risk of developing bronchiectasis. Patients are also prone to develop hematological and skin cancers. Here, we present the case of a patient, the only child of apparently unrelated Serbian parents, affected by PN resulting from the homozygous mutation NM_024598.3:c.243G>A (p.Trp81Ter) of *USB1*; early onset of poikiloderma (1 year of age) was associated with cutaneous mastocytosis. We also provide a review of the literature on this uncommon condition with a focus on dermatological findings.

## Introduction

Poikiloderma with neutropenia (PN) is a very rare genetic disorder with few cases reported worldwide (1) since its first description in 1991 by Clericuzio et al. (2). PN mainly affects the skin with poikiloderma, hyperkeratotic nails, generalized hyperkeratosis on palms and soles, and neutropenia. Other less frequent features may be present including short stature, recurrent pulmonary infections, hypogonadotropic hypogonadism, dysmorphisms, hepatosplenomegaly, and laboratory abnormalities (1). Prognosis strongly depends on recurrent infections and on the risk of developing hematological and skin cancers. Cancer has a significant impact on mortality. Ninety percent of patients with PN have recurrent respiratory issues. Children are typically affected by recurrent otitis media and sinusitis. Several lung conditions can be observed, including infections, bronchiectasis, abscesses, and granulomas, which severely impact on morbidity. Moreover, although frequency of infections reduces dramatically after the first decade, bronchiectasis, productive cough, and reactive airway disease remain during adulthood (1).

Given the extreme rarity of the disease, clinical history and uncommon dermatological findings are still poorly understood. The clinical heterogeneity of PN and why some patients are more prone to develop cancer than others still need to be elucidated.

## Case Report

**Figure 1:**
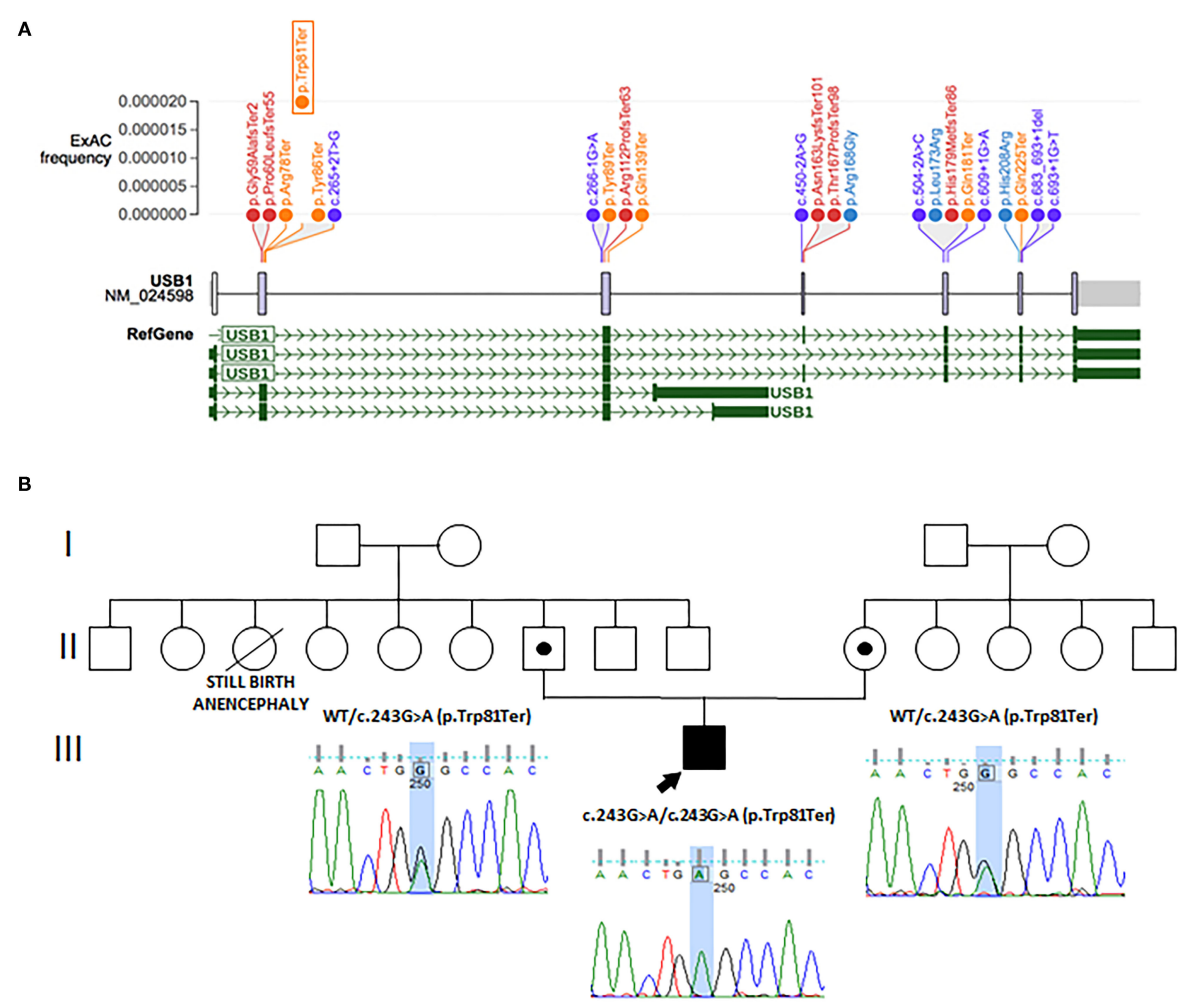
**(A)** Schematic representation of intragenic mutations of *USB1*. Nonsense variants are in orange, frameshift in red, splicing in violet, and missense variants in blue. Protein domains are in gray and refer to HVSL, uncharacterized conserved protein. All variants were extrapolated from ClinVar (RefSeq NM_024598.3). They are distributed according to their ExAc frequency (gnomad.broadinstitute.org). The mutation identified in our proband is framed. **(B)** Family tree, with electropherogram results of affected patient and carriers.

A 6-month-old male baby was referred to our pediatric unit for evaluation of an unusual appearance of his skin. The patient was subsequently referred on to our pediatric hematology unit for severe neutropenia and history of recurrent pneumonia. He was born to young and healthy nonconsanguineous parents after an uneventful pregnancy (Figure 1B).

Parents originated from two small villages in Serbia, only 10 km distant. The child was born at 32 weeks because of premature rupture of membranes, with a neonatal weight of 1,990 g (0.7 SD), length of 45 cm (1.12 SD), and cranial circumference of 29.5 cm (−0.3 SD). He suffered from neonatal hypocalcemia and icterus and was discharged after 21 days. At birth, an atrial septal defect (patent foramen ovale) was documented.

At 30 days of life, the patient was hospitalized because of cyanosis; he received a diagnosis of sepsis and was treated with intravenous infusion of amoxicillin/clavulanic acid and netilmicin. Neutropenia was noted (white cell count, 5,190; neutrophils, 360). At 4 months of life, he developed bronchiolitis and, soon after, was referred to our hematology unit due to persistence of neutropenia. At that time, splenomegaly was already present. Following Italian guidelines, he underwent several tests to exclude postinfection, drug-induced, autoimmune, and cyclic neutropenia (3). Bone marrow examination showed no arrest of myelopoiesis maturation at the promyelocyte stage (a marker of severe congenital neutropenia) and no infiltration by leukemic or Gaucher cells. Dermatitis was noted at around 5 months and progressively worsened, causing the child to be referred to our dermatology unit.

**Figure 2:**
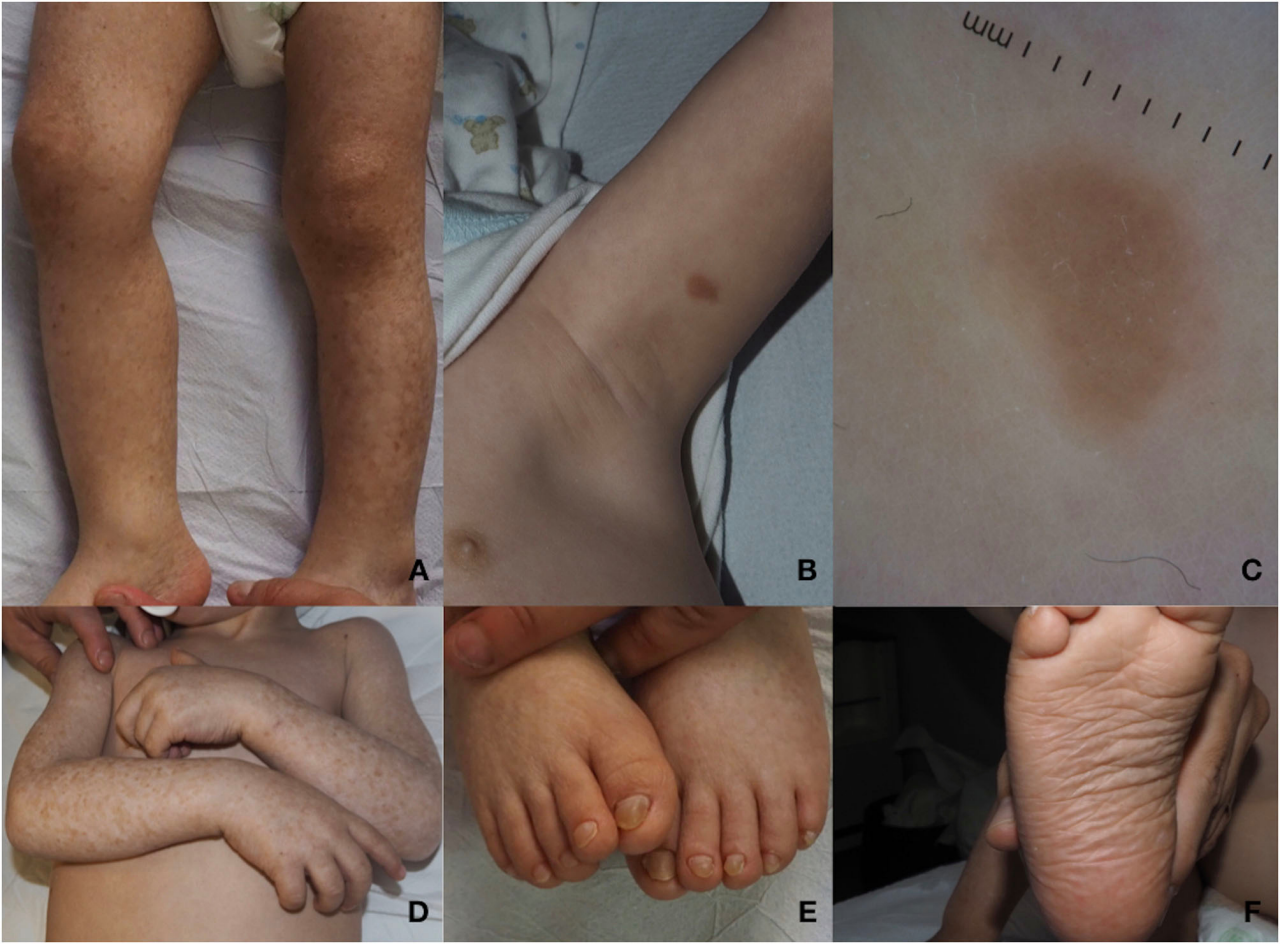
**(A)** Diffuse reticulated squamous hyperpigmentation surrounding slightly hypopigmented areas of the lower limbs. **(B)** Brownish infiltrated plaque on the left arm. **(C)** On polarized light dermoscopy, a homogeneous light brown structureless area is observed. **(D)** Mottled hypo- and hyperpigmentation on the arms, lesions typically localized on photoexposed areas. **(E)** Pachyonychia of the toenails. **(F)** Plantar spiny keratoderma.

Dermatological examination showed diffuse reticulate scaly hyperpigmentation surrounding multiple slightly hypopigmented areas (Figures 2A,D). On the trunk and on the left arm, two brownish infiltrated papules were observed (Figure 2B). Polarized light dermoscopic examination revealed homogeneous light brown structureless areas (Figure 2C). Pachyonychia of the toenails and plantar spiny keratoderma were also present (Figures 2E,F).

**Figure 3:**
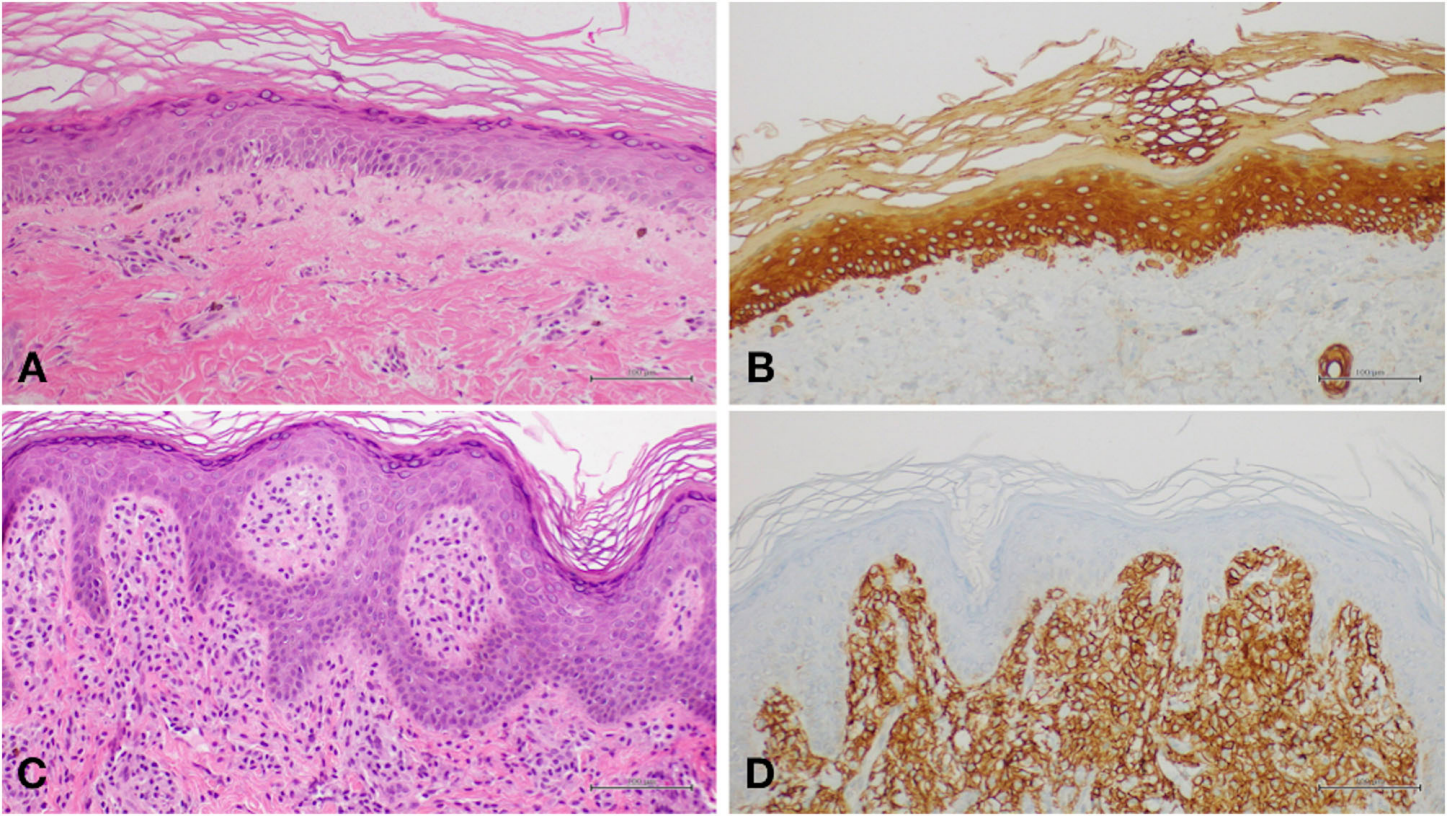
**(A)** Histology of poikiloderma showing slightly atrophic and flattened epidermis, focal images of hydropic degeneration of the basal layer, light perivascular lymphohistiocytic infiltrate, and pigmentary incontinence. **(B)** Several Civatte bodies highlighted by high molecular weight cytokeratin (HMW-CK) immunostaining. **(C)** In mastocytoma, histological examination showed a dense cellular infiltrate occupying the superficial dermis. The cells were characterized by moderately abundant, amphophilic, and finely granular cytoplasm and oval-shaped nuclei with clumped chromatin. **(D)** Immunohistochemical investigation showing positivity for CD117 (c-Kit).

On the basis of clinical appearance and history, a provisional diagnosis of PN was suspected. Two biopsy specimens were obtained, one from reticulate hyperpigmentation on the lower limbs and another from the brownish macule on the left arm. Histopathology on the first specimen confirmed the diagnosis of poikiloderma, showing slightly atrophic and flattened epidermis, focal images of hydropic degeneration of the basal layer, light perivascular lymphohistiocytic infiltrate, and pigmentary incontinence (Figure 3A). Several Civatte bodies were present, as highlighted by high molecular weight cytokeratin (HMW-CK) immunostaining (Figure 3B). The second specimen showed histological features compatible with the diagnosis of cutaneous mastocytosis, including a dense cellular infiltrate occupying the superficial dermis; the cells were characterized by moderately abundant, amphophilic, and finely granular cytoplasm and oval-shaped nuclei with clumped chromatin; immunohistochemical investigation demonstrated positivity for CD117 (c-Kit) (Figures 3C,D). At 8 months of age, a motor developmental delay was diagnosed, and psychomotor rehabilitation was started. The infant was able to sit by 9 months and walk by 18 months. He has been presenting recurrent respiratory infections (mainly involving the upper airways), persistent mild elevation of transaminases, and splenomegaly.

The child received anti-flu vaccination at around 2.6 years of age without any collateral effect. Recently, at the age of 2.8 years, he had coronavirus disease 2019 (COVID-19) with very mild flu-like symptoms and without complications. One month after a negative molecular test result for severe acute respiratory syndrome coronavirus 2 (SARS-CoV-2), he received the measles, mumps, and rubella (MMR) vaccine. Five days later, he was admitted because of fever (T_max_ = 39). Hyperferritinemia (3,336 ng/ml), slightly high C-reactive protein (2.31 mg/dl), and neutrophil count reaching 2,000 cell/L were documented. Thoracic radiography excluded pneumonia, abdominal ultrasound simply confirmed the previously noted splenomegaly, urine culture was negative, and procalcitonin value was normal. He was treated with intramuscular ceftriaxone for 5 days and subsequently with oral cephalosporin for 5 more days, with resolution of fever 36 h after the first antibiotic dose. After 10 days of antibiotic therapy, ferritin had decreased to 750 ng/ml and neutrophil count to 600 cell/L. At the last examination, at 2 years and 10 months, his weight was 11.6 kg (−1.5 SD), height was 90 cm (−1.3 SD), and cranial circumference was 49 cm (just below the median value), compatible with a growth delay. Speech was still absent.

Clinical exome analysis on the family trio detected in the proband a homozygous nonsense variant [NM_024598.3: c.243G>A; p.(Trp81Ter)] in *USB1*, the gene encoding for the U6 small nuclear RNA (snRNA) phosphodiesterase 1, whose mutations cause PN (MIM 604173). This variant is already reported as pathogenic (ClinVar ID 156347) (4), and segregation analysis confirmed that both parents were heterozygous for this variant, suggesting geographical consanguinity (Figure 1B). No other pathogenic or likely pathogenic variants, possibly related to the patient's phenotype, were found in other genes.

## Discussion

PN (namely Clericuzio-type poikiloderma) is an extremely rare autosomal recessive disease resulting from mutations in *USB1* and characterized by a combination of poikiloderma and neutropenia, mainly causing severe respiratory infections. Poikiloderma occurs early in childhood, presenting with the typical features of a reticulated erythema and hyperpigmentation-delimiting hypopigmented atrophic areas. While poikiloderma predisposes to the rare occurrence of skin cancer, respiratory infections and bronchiectasis may be life threatening and lead to a poor prognosis. Patients are prone to develop myelodysplastic syndrome and, rarely, acute myelogenous leukemia in adult age. A wide spectrum of other signs may be observed in PN including hypogonadotropic hypogonadism, osteopenia, hepatosplenomegaly, and other uncommon features (1, 2, 4–17).

Here, we describe the onset and clinical history of PN, providing useful information for clinicians facing this very rare disease. Our case fits the typical PN phenotype. As expected, postnatal growth delay was present (1). Neutropenia occurred in the first year of life and was due to dysmyelopoiesis only affecting the granulocyte lineage. The patient also presented mild developmental delay likely related to his perinatal history and suspected hypoxic brain damage rather than the PN diagnosis. While some degree of early developmental delays are reported, intellectual disability has not in fact been associated with PN. Congenital heart defects are not usually present in PN. One patient described by Colombo et al. (8) presented pulmonic stenosis and prolonged QT at ECG; the authors reported a congenital heart defect in another child although without specifying the type. Whether heart findings are incidental or associated with PN needs to be elucidated. In any event, no patients carrying the c.243G>A variant had heart problems. We observed mild, yet persistent elevation of aminotransferases and ferritin as well as splenomegaly and recurrent mild airway infections. Observed facial somatic features of patients with PN included prominent forehead, depression of nasal bridge, midfacial retrusion, and sparse eyelashes and eyebrows. Our patient does not present any particular dysmorphism.

Cutaneous manifestations are among the most common signs in PN, with poikiloderma itself being the defining feature of the disease. In addition to poikiloderma, extensively described above, other cutaneous presentations can be observed in PN, such as photosensitivity, pachyonychia, keratoderma of palms/soles, calcinosis cutis, and atrophic plaques (1). In particular, photosensitivity manifests as higher susceptibility to development of acute and chronic damage following sun exposure, thus necessitating the use of sunscreens and physical protections in patients with PN (1). Pachyonychia and palmoplantar keratoderma refer to thickening of nail plates and of the skin of palms/soles, respectively. Both are observed in various skin conditions and cannot be considered specific to PN. Finally, calcinosis cutis and atrophic plaques may be regarded as a direct consequence of poikiloderma (1). Specifically, calcinosis cutis presents with small firm papules or nodules located on elbows, knees, and pinnae, or, rarely, more diffusely.

Patients with PN are also prone to development of bacterial and dermatophyte skin infections (1). Cellulitis is occasionally observed in adolescents with secondary non-healing ulcers, which sometimes spontaneously develop without being preceded by skin infection (1).

As regards the risk of skin cancer, a few reports have described the occurrence of squamous cell carcinoma (SCC) (16, 17) in young patients with PN. In other syndromes associated with poikiloderma (e.g., xeroderma pigmentosum), the early occurrence of skin cancer including SCC, basal cell carcinoma, and melanoma is quite common.

Fewer than 30 pathogenic mutations, both private and recurrent variants, have been found in PN (Figure 1A). All seem to lead to a loss-of-function effect. The c.243G>A, p.(Trp81Ter) variant found in our case was already reported in at least three northern European families (4, 18–20). Colombo et al. (21) reviewed cancer risk in six patients with the c.243G>A, p.(Trp81Ter) variant reported until 2018, one of whom originally described by the authors. They showed that c.243G>A is one of the three mutations, together with c.531delA, p.(His179MetfsTer86) and c.541C>T, p.(Gln181Ter), associated with a higher risk of developing cancer (1). We reviewed the phenotypes of patients carrying the c.243G>A variant reported up to the present date, including both homozygous and compound heterozygous mutations (Table 1). We found at least five patients harboring the c.243G>C variant, four in the homozygous state and one in a compound heterozygous state with c.541C>T. The latter mutation also leads to a premature stop codon. A complete loss of protein expression is expected in all these patients. The slight phenotypic variability observed in all patients (i.e., in terms of growth delay, dysmorphic features) might be related to other concurrent genetic or environmental factors.

**Table 1:** Revision of cases with the c.243G>A mutation reported at today together with the patient here reported.

	**Porter et al. (20) and Walne et al. (18)**		**Arnold et al. (4)**	**Piard et al. (19)**	**Colombo et al. (21)**	**Current study**	**Proportion/total**	**Percentage**
**Patient ID**	**RT1 II-1**	**RT1 II-2**	**Patient 1**	**c16-01**	**#48**	**Our patient**		
*USB1* genotype	c.[243G>A];[243G>A]	NA yet older sister of the RT1 II-1	c.[243G>A];[243G>A]	c.[243G>A]; c.[267T>A]	c.[243G>A];[541C>T]	c.[243G>A];[243G>A]	–	–
Protein effect	p.(Trp81Ter)		p.(Trp81Ter)	p.(Trp81Ter)	p.(Trp81Ter); p.(Gln181Ter)	p.(Trp81Ter)	–	–
Age at evaluation/gender	21 years/F	NA/F	4 years/M	4.5 years/F	36 years/M	3 years/M	3F/2M	60%/40%
**Key features**								
Poikiloderma	+	+	+	+	+	+	6/6	100%
Persistent neutropenia	+	NA	+	+	+	+	5/5	100%
Recurrent infections	+	+	+	–	+	+	6/6	100%
Palmoplantar keratoderma	?+	NA	+	+	+	+	5/5	100%
Pachyonychia of the great toenails	+	+	+	+	+	+	6/6	100%
Photosensitivity	+	NA	+	+	+	+	5/5	100%
**Additional features**								
Hepatosplenomegaly	**–**	NA	+	**–**	**–**	**+**	2/5	40%
Craniofacial dysmorphisms	+	NA	**–**	**–**	+	**–**	2/5	40%
Non-descended or retractile testes	not applicable	NA	+	Not applicable	**–**	**–**	1/3	33%
Thin/sparse hair	**–**	NA	**–**	**–**	+	+	2/5	40%
Dental caries/defects	**–**	NA	**–**	+	+	+	3/5	60%
Growth retardation/short stature	+	NA	+	**–**	+	+	4/5	80%
Thrombocytopenia	**–**	NA	+ transitory	**–**	**–**	**–**	1/5	20%
Leucopenia	+	NA	**–**	+	+	+	3/5	60%
Elevated lactate dehydrogenase	NA	NA	+	+	**–**	+	3/4	75%
Elevated ferritin	NA	NA	+	+	**–**	+	3/4	75%
Appropriate rise in neutrophil numbers during episodes of infection	+	NA	+	**–**	**–**	+	3/5	60%
Bone marrow evaluation	Hypocellularity	AML	NA	NA	Hypocellularity	Normal	–	–
Bone abnormalities	Osteosclerosis	NA	NA	NA	Osteopenia sclerosis of distal phalanges	NA	2/2	100%
Others	Skin cancer	Deceased because of AML			Hemolytic anemia; Hypotonia; hypogonadism	Motor and speech delay, patent foramen ovale		

*NA, not available; F, female; M, male; AML, acute myeloid leukemia; ASD, atrial septal defect*.

Mastocytosis is a complex disease with a large spectrum of clinical manifestations caused by the proliferation and infiltration of benign mast cells in different tissues. Cutaneous mastocytosis (CM) is a common type of mastocytosis, mostly seen in children, with a broad variety of clinical findings, ranging from more frequent cutaneous maculopapular mastocytosis and mastocytoma to rarer telangiectasia macularis eruptiva perstans and diffuse mastocytosis (22). The incidence of CM is not precisely known, but in the one and only study available, it was found to be 1 per 500 children presenting to pediatric dermatology clinics (23). CM has an indolent course in childhood and can be considered a benign disease with exceptional systemic signs or symptoms, such as gastrointestinal and cardiovascular symptoms mostly caused by abundant histamine release by mast cells. Due to the hyperproduction of histamine, children with CM experience cutaneous manifestations, including mainly pruritus, erythema, red dermographism, flushing, hives, and Darier's sign, namely, the enlargement and swelling (rarely blistering in the first months of life) of lesions after induced or spontaneous rubbing. Serum tryptase levels may be considered a good marker of systemic involvement of mastocytosis, with a threshold of 20 ng/ml. Prognosis of CM is good, as the disease usually spontaneously resolves by adolescence or adulthood. Treatment with antihistamines and corticosteroids may be necessary for symptom control, and educational information could be useful in limiting Darier's sign elicitation and excessive histamine release (22).

The association between PN and CM has never been reported before in the literature and could be considered casual, given the relatively common occurrence of mastocytosis in children. It could be argued that a subclinical immunodeficiency might be a triggering factor of mastocytosis in our patient, although this hypothesis seems highly unlikely, as most pediatric mastocytoses are not secondary to immunodeficiency but often to *KIT* mutations found outside exon 17. Apart from the Clericuzio type of PN, no other forms of syndromic poikiloderma have been associated with mastocytosis. On this basis, we are confident that in our patient, the association was likely coincidental. Notably, bone marrow biopsy excluded a systemic involvement of mastocytosis, which was in any event highly improbable given the patient's age and his low levels of serum tryptase.

In previously reported cases carrying the c.243G>A mutation in *USB1*, poikiloderma, persistent neutropenia, recurrent infections, palmoplantar keratoderma, pachyonychia of the great toenails, and photosensitivity are constant features, as expected (Table 1). Elevation of lactate dehydrogenase (LDH) and ferritin levels are also frequent (75% of cases), followed by dental problems and growth failure or short stature. It is worth reporting that neutrophil values appropriately rise (although without achieving levels higher than normal) during infections in at least 60% of patients. Mechanisms leading to this phenomenon should be further studied in order to identify potential molecular pathways and targeted drugs able to increase neutrophil count and hopefully reduce the frequency and severity of infectious diseases. Two sisters described by Porter et al. (20) were diagnosed with cancer; almost all the other cases were very young at the time of presentation. Thus, the risk of developing cancer still needs to be quantified in all such patients.

Clinicians should be aware of the existence of PN when facing a precocious isolated neutropenia, even before typical dermatological signs become evident. A multidisciplinary approach including at least dermatologists, pediatricians, and geneticists is strongly advised for the management of the complex clinical scenario in patients with PN.

## Data Availability Statement

The original contributions generated for the study are included in the article, further inquiries can be directed to the corresponding author/s.

## Ethics Statement

Ethical review and approval was not required for the study on human participants in accordance with the local legislation and institutional requirements. Written informed consent to participate in this study was provided by the participants' legal guardian/next of kin. Written informed consent was obtained from the minor(s)' legal guardian/next of kin for the publication of any potentially identifiable images or data included in this article.

## Author Contributions

VP, TR, and CS: conception, design, and draft of the work. DP, EP, and MD: acquisition of data and draft of the work. GP: molecular analysis and draft of the work. AR and ED: acquisition of data and preparation of figures. GA: critical revision of the work for important intellectual content. All authors approved the submitted version of the paper and agree for publication of the content and also agree to be accountable for all aspects of the work in ensuring that questions related to the accuracy or integrity of any part of the work are appropriately investigated and resolved.

## Conflict of Interest

The authors declare that the research was conducted in the absence of any commercial or financial relationships that could be construed as a potential conflict of interest.
